# *RPA3-UMAD1* rs12702634 and rheumatoid arthritis–associated interstitial lung disease in European ancestry

**DOI:** 10.1093/rap/rkae059

**Published:** 2024-06-04

**Authors:** Pierre-Antoine Juge, Jeffrey A Sparks, Steven Gazal, Esther Ebstein, Raphaël Borie, Marie-Pierre Debray, Caroline Kannengiesser, Gregory C McDermott, Jing Cui, Keigo Hayashi, Tracy J Doyle, Coline H M van Moorsel, Joanne J van der Vis, Jan C Grutters, Rachel Knevel, Sascha L Heckert, Eirini Vasarmidi, Katerina M Antoniou, Annette H M van der Helm van Mil, Catherine Boileau, Bruno Crestani, Philippe Dieudé

**Affiliations:** Université de Paris Cité, INSERM UMR 1152, Paris, France; Service de Rhumatologie, Hôpital Bichat-Claude Bernard, AP-HP, Paris, France; Division of Rheumatology, Inflammation, and Immunity, Brigham and Women’s Hospital, Boston, MA, USA; Harvard Medical School, Boston, MA, USA; Department of Population and Public Health Sciences, Keck School of Medicine, University of Southern California, Los Angeles, CA, USA; Center for Genetic Epidemiology, Keck School of Medicine, University of Southern California, Los Angeles, CA, USA; Service de Rhumatologie, Hôpital Bichat-Claude Bernard, AP-HP, Paris, France; Université de Paris Cité, INSERM UMR 1152, Paris, France; Service de Pneumologie, Hôpital Bichat-Claude Bernard, AP-HP, Paris, France; Service de Radiologie, Hôpital Bichat-Claude Bernard, AP-HP, Paris, France; Université de Paris Cité, INSERM UMR 1152, Paris, France; Service de Génétique, Hôpital Bichat-Claude Bernard, AP-HP, Paris, France; Division of Rheumatology, Inflammation, and Immunity, Brigham and Women’s Hospital, Boston, MA, USA; Harvard Medical School, Boston, MA, USA; Division of Rheumatology, Inflammation, and Immunity, Brigham and Women’s Hospital, Boston, MA, USA; Harvard Medical School, Boston, MA, USA; Division of Rheumatology, Inflammation, and Immunity, Brigham and Women’s Hospital, Boston, MA, USA; Harvard Medical School, Boston, MA, USA; Harvard Medical School, Boston, MA, USA; Division of Pulmonary and Critical Care, Brigham and Women's Hospital, Boston, MA, USA; Interstitial Lung Disease Center of Excellence, Department of Pulmonology, St Antonius Hospital, Nieuwegein, The Netherlands; Division of Heart and Lungs, University Medical Center Utrecht, Utrecht, The Netherlands; Interstitial Lung Disease Center of Excellence, Department of Pulmonology, St Antonius Hospital, Nieuwegein, The Netherlands; Division of Heart and Lungs, University Medical Center Utrecht, Utrecht, The Netherlands; Interstitial Lung Disease Center of Excellence, Department of Pulmonology, St Antonius Hospital, Nieuwegein, The Netherlands; Division of Heart and Lungs, University Medical Center Utrecht, Utrecht, The Netherlands; Department of Rheumatology, Leiden University Medical Center, Leiden, The Netherlands; Translational & Clinical Research Institute, Newcastle University, UK; Department of Rheumatology, Leiden University Medical Center, Leiden, The Netherlands; Laboratory of Molecular and Cellular Pneumonology, Department of Respiratory Medicine, School of Medicine, University of Crete, Heraklion, Greece; Laboratory of Molecular and Cellular Pneumonology, Department of Respiratory Medicine, School of Medicine, University of Crete, Heraklion, Greece; Department of Rheumatology, Leiden University Medical Center, Leiden, The Netherlands; Department of Rheumatology, Erasmus MC, Rotterdam, The Netherlands; Service de Génétique, Hôpital Bichat-Claude Bernard, AP-HP, Paris, France; Université de Paris Cité, INSERM UMR 1152, Paris, France; Service de Pneumologie, Hôpital Bichat-Claude Bernard, AP-HP, Paris, France; Université de Paris Cité, INSERM UMR 1152, Paris, France; Service de Rhumatologie, Hôpital Bichat-Claude Bernard, AP-HP, Paris, France

**Keywords:** rheumatoid arthritis, interstitial lung disease, genetic

## Abstract

**Objective:**

Recently, a genome-wide association study identified an association between RA-associated interstitial lung disease (ILD) and *RPA3-UMAD1* rs12702634 in the Japanese population, especially for patients with a usual interstitial pneumonia (UIP) pattern. We aimed to replicate this association in a European population and test for interaction with *MUC5B* rs35705950.

**Methods:**

In this genetic case–control association study, patients with RA and ILD and controls with RA and no ILD were included from France, the USA and the Netherlands. Only cases and controls from European genetic ancestries determined by principal components analysis were included in the analyses. RA was defined by the 1987 ACR or 2010 ACR/EULAR criteria and ILD by chest high-resolution CT scan, except in the control dataset from the Netherlands, where the absence of ILD was determined by chart review. Patients were genotyped for *RPA3-UMAD1* rs12702634 and *MUC5B* rs35705950. Associations were tested using logistic regression adjusted for sex, age at RA onset, age at ILD onset or at certified absence of ILD, tobacco smoking status and country of origin.

**Results:**

Among the 883 patients included, 322 were RA-ILD cases (36.5%). *MUC5B* rs35705950 was strongly associated with RA-ILD in all datasets {combined adjusted odds ratio [OR] 2.9 [95% CI 2.1, 3.9], *P* = 1.1 × 10^−11^. No association between *RPA3-UMAD1* rs12702634 and RA-ILD was observed [combined OR 1.2 (95% CI 0.8, 1.6), *P* = 0.31. No interaction was found between *RPA3-UMAD1* rs12702634 and *MUC5B* rs35705950 (*P* = 0.70).

**Conclusion:**

Our findings did not support a contribution of *RPA3-UMAD1* rs12702634 to the overall RA-ILD susceptibility in the European population.

Key messages

- The association between *RPA3-UMAD1* rs12702634 and RA-ILD was not replicated in this European genetic association study.
- No interaction was found between *RPA3-UMAD1* rs12702634 and *MUC5B* rs35705950.
- Larger studies are needed to fully exclude a modest contribution of *RPA3-UMAD1* rs12702634 to the risk of ILD in patients with RA.

## Introduction

Interstitial lung disease (ILD) is a severe extra-articular manifestation in 10–60% of patients with RA [1–4]. The genetic background of RA-ILD has recently been investigated, providing evidence for the contribution of *MUC5B* rs35705950, a common variant previously associated with idiopathic pulmonary fibrosis (IPF), in RA-ILD susceptibility [5]. *MUC5B* rs35705950 is the strongest genetic risk factor for RA-ILD in the European population, the association being restricted to patients with RA-ILD with a definite or probable usual interstitial pneumonia (UIP) pattern on chest high-resolution CT (HRCT) scans [5]. *MUC5B* rs35705950 is a functional variant, the T risk allele being associated with an increased expression of mucin-5b, the protein encoded by *MUC5B* [6]. Outside *MUC5B*, rare variants in telomere-related genes have been suggested to be more frequently observed in RA-ILD [7].

Recently, a genome-wide association study (GWAS) was performed in three independent Japanese datasets of patients with RA with and without ILD. This study identified a strong association between the *RPA3-UMAD1* rs12702634 variant and RA-ILD [odds ratio (OR) 2.04 (95% CI 1.59, 2.60), *P* = 1.5 × 10^−8^] [8]. The association was stronger for patients having a UIP or probable UIP chest HRCT pattern [8]. rs12702634 has been reported to affect RPA3 expression levels, implying *RPA3* is a candidate risk gene in the locus. Of interest, RPA3 is a subunit of RPA that plays an essential role in DNA replication and has a potential role in telomere maintenance [9]. Conversely, an independent Japanese study failed to replicate these findings [10]. Further replication in other populations is therefore needed to further conclude the exact contribution of the *RPA3-UMAD1* rs12702634 variant in RA-ILD susceptibility.

The objective of this study was to test for replication in a European population the association between RA-ILD and *RPA3-UMAD1* rs12702634, notably in patients with a UIP or probable UIP chest HRCT pattern and to investigate a potential interaction between *RPA3-UMAD1* rs12702634 and *MUC5B* rs35705950.

## Methods

### Patients

For this case–control study, patients with RA-ILD (cases) and without ILD (controls) were included from three different countries (France, USA and the Netherlands). The French dataset consisted of patients from the French RA-ILD network [7]. Patients from the US dataset were recruited from the Mass General Brigham Biobank (Boston, MA, USA) as previously described [11]. Patients from the Netherlands were included from the St Antonius ILD expert centre for the RA-ILD population and from the Leiden early arthritis cohort for the control population [12]. All patients met the ACR 1987 and/or ACR/EULAR 2010 RA criteria according to chart review. In the French and the US datasets, the ILD status was defined using a chest HRCT scan reviewed by expert readers from each centre for both cases and controls. In the dataset from the Netherlands, only cases were determined by chest HRCT scan, whereas the absence of ILD was determined by chart review. For the three datasets, an ILD chest HRCT scan pattern (i.e. UIP *vs* non-UIP) was classified using international recommendations by the same expert readers from each centre who reviewed chest HRCT scan imaging [13]. The institutional review board at each institution approved all protocols and all patients provided written informed consent (Northern and Western French Ethic Committee III no. 2019-31 for France, Mass General Brigham Institutional Review Board no. 2019P000264 for the US dataset, Medical Ethics Committee of St Antonius Hospital no. R05-08A for the cases from the Netherlands and the regional ethics committee at Leiden University Medical Center for the controls in the Netherlands).

### Genotyping

All patients were genotyped for *RPA3-UMAD1* rs12702634, *MUC5B* rs35705950 and 24 ancestry-informative markers (AIMs). An allele-specific PCR system was used for the French population and the patients from St Antonius (KASPAT genotyping, LGC Genomic, Berlin, Germany). Genotypes for the patients from Mass General Brigham were directly obtained using the Multi-Ethnic Genotyping Array (MEGA), Expanded Multi-Ethnic Genotyping Array (MEGA^EX^) (both from Illumina, San Diego, CA, USA) or imputed the using the Michigan imputation server with the Haplotype Reference Consortium (HRC) as the reference panel. The allele-specific PCR system was used for genotyping some Mass General Brigham patients who had banked DNA but no genetic data from the MEGAs. Controls from Leiden were genotyped using the Illumina GSA platform with 750 000 or 250 000 single nucleotide polymorphisms (SNPs) or imputed using the Michigan imputation server with the HRC as the reference panel.

### Statistical analyses

Genetic association analyses included only patients with European ancestry assessed by a principal components analysis (PCA) using 24 AIMs. Characteristics of cases and controls were first compared using a univariate logistic regression for descriptive interest. Associations between RA-ILD and *RPA3-UMAD1* rs12702634 and *MUC5B* rs35705950 were tested using a multivariable logistic regression adjusted for sex, age at RA onset, age at index date (date of ILD diagnosis for RA-ILD cases, date of the last normal chest HRCT scan for controls from France and the USA and date of record review for controls from the Netherlands), smoking status (ever *vs* never smokers) and country of origin (adjusted results). Interaction between *RPA3-UMAD1* rs12702634 and *MUC5B* rs35705950 was tested according to the significance of the interaction term in the logistic regression model. All statistical analyses used R version 4.2.2 (R Foundation for Statistical Computing, Vienna, Austria).

## Results

### Sample characteristics

There were a total 1073 patients with RA recruited for this study and we analysed 883 (322 RA-ILD cases and 561 controls) that had European ancestries by PCA. Characteristics of the 883 analysed patients are summarized in Table 1. Briefly, 275 were male (31.1%) and the mean age at RA onset was 52.1  years (s.d. 14.4). When compared with controls, cases with RA-ILD were more frequently male (46.0% *vs* 22.7%) and had an older age at RA onset [55.0 years (s.d. 13.0) *vs* 47.3 years (s.d. 14.4)]. Among cases with RA-ILD, 125 (50.2%) had RA-UIP. The minor allele frequencies (MAFs) of *MUC5B* rs35705950 and *RPA3-UMAD1* rs12702634 are shown in Table 1. Descriptive comparison of cases and controls included in the analyses are available in Supplementary Table S1, available at *Rheumatology Advances in Practice* online. Based on the previously reported MAF of *RPA3-UMAD1* rs12702634 and an OR of 2.04 [8], our sample size provided a power of detection >95%.

**Table 1.** Characteristics of RA-ILD cases and controls with European ancestry{start}

Characteristics	Combined		France		US		The Netherlands	
	RA-ILD cases (*n* = 322)	Controls (*n* = 561)	RA-ILD cases (*n* = 192)	Controls (*n* = 256)	RA-ILD cases (*n* = 73)	Controls (*n* = 213)	RA-ILD cases (*n* = 57)	Controls (*n* = 92)
Male, *n* (%)	148 (46.0)	127 (22.7)	87 (45.3)	55 (21.5)	25 (34.2)	52 (24.4)	36 (63.2)	20 (21.7)
Ever smoker, *n* (%)	209 (66.3)	310 (56.2)	115 (61.5)	130 (52.2)	50 (68.5)	126 (59.4)	44 (80.0)	54 (59.3)
Age at RA onset, years, mean (s.d.)	55.0 (13.0)	47.3 (14.4)	55.0 (13.7)	44.0 (12.8)	53.0 (12.0)	50.2 (15.8)	57.3 (11.8)	49.7 (13.1)
Age at index date,^a^ years, mean (s.d.)	63.3 (10.6)	61.2 (13.1)	63.6 (10.0)	57.4 (12.5)	61.5 (11.7)	61.8 (12.1)	64.5 (10.5)	70.2 (12.3)
RA duration at index date,^a^ years, mean (s.d.)	8.5 (10.5)	13.9 (10.5)	8.8 (10.7)	13.6 (8.3)	8.5 (11.3)	11.6 (12.8)	7.4 (8.8)	20.6 (6.3)
UIP, *n* (%)	143 (49.3)	–	97 (59.1)	–	18 (26.1)	–	28 (49.1)	–
*MUC5B* rs35705950, MAF	0.27	0.10	0.28	0.12	0.25	0.09	0.24	0.07
*RPA3-UMAD1* rs12702634, MAF	0.14	0.12	0.12	0.13	0.15	0.13	0.17	0.07

a Index date is the date of ILD diagnosis for RA-ILD cases, the date of the last normal chest HRCT scan for controls from France and the USA or the date of record review for controls from the Netherlands.

### Genetic associations with RA-ILD

The *MUC5B* rs35705950 T risk allele was strongly associated with RA-ILD in all three datasets with a combined OR of 2.8 (95% CI 2.1, 3.8; *P* = 5.8 × 10^−11^) (Table 2). In the three different datasets, no association was found between *RPA3-UMAD1* rs12702634 and RA-ILD [OR 0.9 (95% CI 0.6, 1.5), *P* = 0.81 in the French dataset, OR 1.2 (95% CI 0.7, 2.1), *P* = 0.53 in the USA dataset and OR 2.1 (95% CI 0.6, 7.6), *P* = 0.24 in the Netherlands dataset], resulting in a combined OR of 1.2 (95% CI 0.8, 1.6; *P* = 0.31) (Table 2). No interaction between *MUC5B* rs35705950 and *RPA3-UMAD1* rs12702634 was observed (*P* = 0.70).

**Table 2.** Results of the genetic case–control association studies

Genetic association	France		US		The Netherlands		Combined	
	aOR (95% CI)	*P*-value	aOR (95% CI)	*P*-value	aOR (95% CI)	*P*-value	aOR (95% CI)	*P*-value
RA-ILD *vs* control								
*MUC5B* rs35705950	2.6 (1.7, 4.0)	1.0 × 10^−5^	3.3 (1.8, 5.8)	3.1 × 10^−5^	5.5 (1.6, 21.2)	0.008	2.8 (2.1, 3.8)	5.8 × 10^−11^
*RPA3-UMAD1* rs12702634	0.9 (0.6, 1.5)	0.81	1.2 (0.7, 2.1)	0.53	2.1 (0.6, 7.6)	0.24	1.2 (0.8, 1.6)	0.31
Interaction		0.07		0.2		0.99		0.70
RA-UIP *vs* control								
*MUC5B* rs35705950	3.6 (2.2, 6.1)	1.2 × 10^−6^	6.9 (2.8, 18.9)	5.3 × 10^−5^	6.1 (1.4, 32.6)	0.02	4.4 (3.0, 6.6)	7.7 × 10^−14^
*RPA3-UMAD1* rs12702634	1.0 (0.6, 1.8)	0.90	1.2 (0.4, 3.0)	0.76	2.2 (0.4, 11.2)	0.33	1.3 (0.9, 1.9)	0.28
Interaction		0.04		0.99		0.99		0.43
Non-UIP RA-ILD *vs* control								
*MUC5B* rs35705950	1.9 (1.1, 3.3)	0.03	2.5 (1.3, 4.8)	0.005	4.2 (0.97, 19.7)	0.06	2.1 (1.4, 3.0)	2.2 × 10^−4^
*RPA3-UMAD1* rs12702634	0.8 (0.3, 1.5)	0.45	0.96 (0.5, 1.9)	0.91	2.4 (0.5, 11.7)	0.26	0.98 (0.6, 1.5)	0.94
Interaction		0.16		0.85		0.99		0.43
RA-UIP *vs* non-UIP RA-ILD								
*MUC5B* rs35705950	1.9 (1.1, 3.7)	0.03	3.1 (1.1, 10.2)	0.04	2.7 (0.9, 8.6)	0.08	2.2 (1.4, 3.5)	6.3 × 10^−4^
*RPA3-UMAD1* rs12702634	1.6 (0.8, 3.8)	0.26	1.2 (0.3, 4.2)	0.80	1.1 (0.3, 4.2)	0.83	1.4 (0.8, 2.4)	0.24
Interaction		0.66		0.42		0.82		0.67

Multivariable results are adjusted for sex, age at RA onset, age at ILD onset or certified absence of ILD, tobacco smoking status and country of origin. They are presented with the *P*-value of the interaction between *MUC5B* rs35705950 and *RPA3-UMAD1* rs12702634.

### Subgroup analyses

Subanalyses performed according to ILD chest HRCT scan patterns found the strongest association between *MUC5B* rs35705950 and RA-UIP compared with non-UIP patterns [combined OR 4.4 (95% CI 3.0, 6.6), *P* = 7.7 × 10^−14^ and OR 2.1 (95% CI 1.4, 3.0), *P* = 2.2 × 10^−4^, respectively], while no association between *RPA3-UMAD1* rs12702634 and RA-UIP or non-UIP RA-ILD was observed [combined analysis OR 1.3 (95% CI 0.9, 1.9), *P* = 0.28 and OR 0.98 (95% CI 0.6, 1.5), *P* = 0.94, respectively] (Table 2). No interaction between *MUC5B* rs35705950 and *RPA3-UMAD1* rs12702634 was observed for RA-UIP and non-UIP RA-ILD (*P* = 0.43 for both) (Table 2). The RA-ILD case-only analysis (i.e. RA-UIP compared with non-UIP RA-ILD) confirmed the stronger contribution of the *MUC5B* rs35705950 variant to the risk of RA-UIP [OR 2.2 (95% CI 1.4, 3.5), *P* = 6.3 × 10^−4^], whereas no difference was observed for *RPA3-UMAD1* rs12702634 [OR 1.4 (95% CI 0.8, 2.4), *P* = 0.24].

Lastly, analyses stratified on smoking status were performed and did not find any association in ever or never smokers, even if these analyses were limited by the relatively small sample size (*n* = 519 for ever smokers and *n* = 348 for never smokers; Supplementary Tables S2 and S3, available at *Rheumatology Advances in Practice* online).

## Discussion

The genetic architecture of RA-ILD is still poorly understood due to the lack of studies with large sample sizes, allowing investigation of rare variants or common variants having weak effects. To date, only *MUC5B* rs35705950, a common variant with a strong effect, has been definitely associated with RA-ILD, with several replication studies from different genetic ancestries [5, 14, 15]. In this study, we did not replicate the possible association of *RPA3-UMAD1* with ILD risk among RA patients with European ancestry.

This is the first replication study testing for association of *RPA3-UMAD1* rs12702634 with RA-ILD in European populations. Although rs12702634 MAFs are comparable between the Japanese and European populations according to gnomAD databases (i.e. MAF *=* 0.12), we did not replicate the significant association with RA-ILD or with RA-UIP that was observed in the Japanese GWAS. Beyond an absence of contribution of *RPA3-UMAD1* rs12702634 to the genetic architecture of the overall RA-ILD, this lack of replication could be for several reasons: genetic heterogeneity across the populations studied, weaker effect size of rs12702634 in European ancestries that could not be captured by our study and a putative role of *RPA3-UMAD1* in the European population driven by a distinct causal variant located in a different haplotype block from the Asian population (i.e. not tagged by rs12702634).

When comparing the statistical analyses performed in the two Japanese studies investigating *RPA3-UMAD1* rs12702634 in RA-ILD, one important difference is that the genetic association analyses were not adjusted for smoking status in the discovery study by Shirai *et al.* [8], whereas smoking history was included as a covariate in the replication study by Higuchi *et al.* [10]. Since tobacco smoking is a known risk factor for RA-ILD, a difference in frequency of ever smokers between cases and controls may have led to a potential bias in the discovery study. As telomerase dysfunction and short telomere length have been associated with several types of ILD, such as IPF and RA-ILD, Shirai *et al.* [8] suggested that *RPA3-UMAD1* rs12702634 could influence ILD risk in RA through the previously reported role of *RPA3* in the regulation of telomerase activity. Following this hypothesis, tobacco smoking is an established risk factors for telomere shortening that could modulate the potential genetic impact of *RPA3-UMAD1* rs12702634 on RA-ILD [16]. In our study, the MAF of *RPA3-UMAD1* rs12702634 was the highest in RA-ILD cases from the Netherlands and no signal was identified in our genetic association analyses when adjusted for smoking status. We also did not find different signals in our subgroup analyses performed according to smoking status, even if the conclusions are limited by relatively small sample size. Due to missing data and small sample size, an analysis of tobacco smoking level could not be performed. A potential association of *RPA3-UMAD1* rs12702634 specific to patients having the highest exposure to tobacco smoking could not be ruled out. Further studies in a larger population are needed to better understand the relationship between *RPA3-UMAD1* rs12702634, tobacco smoking, telomere length and RA-ILD.

Our study was specifically designed to investigate the contribution of rs12702634 to RA-ILD and RA-UIP susceptibility in patients of European ancestry. The 143 patients with RA-UIP included in our combined European datasets provided an adequate power of detection of the association signal identified in the Japanese population [8] (power >90% for an OR of 2.1 estimated from the original Japanese publication and an MAF of 0.14). In line with this, when comparing RA-UIP with non-UIP RA-ILD, no significant increase of frequency of the putative risk allele was observed. Still, our results could derive from a type I error or a potential modest role of *RPA3-UMAD1* rs12702634 in the risk of RA-UIP. Additional studies in larger European populations are therefore needed to clarify if there is genetic heterogeneity regarding the contribution of rs12702634 to RA-ILD susceptibility, notably in the subset of patients with a UIP HRCT pattern.

In addition to the above-mentioned weaknesses, our study had some other limitations to consider. First, due to the retrospective design and missing data, some established risk factors for ILD in RA could not be included in the association analyses, such as seropositivity for RF or ACPAs or RA activity. Second, in the Dutch dataset, ILD absence was determined by medical review and not chest HRCT, leading to a potential misclassification of undiagnosed subclinical RA-ILD cases into the control group. However, RA-ILD phenotypes were defined by HRCT for most of the patients [791/883 (89.6%)] and we were able to replicate the association with MUC5B rs35705950, and identify other reported risk factors for RA-ILD when comparing RA-ILD cases and controls (i.e. male sex, older age at RA onset and ever smoking), supporting a low impact of potential confounders.

Unexpectedly, RA duration was longer in the control groups. However, conflicting results have been published regarding the association between RA duration and ILD in RA, partly because of a complex interaction with age and RA onset [17]. Moreover, the design of our study (i.e. retrospective case–control association study) does not allow a correct analysis of such a variable.

In conclusion, our findings did not support a contribution of *RPA3-UMAD1* rs12702634 to overall RA-ILD susceptibility in the European population. These results highlight the importance of independent replication studies and promote future multi-ancestry Genome-Wide Association Studies dedicated to RA-ILD.

## Supplementary Material

rkae059_Supplementary_Data

## Data Availability

Data are available upon request.
