# Supplementary material for: RPA3-UMAD1 rs12702634 and rheumatoid arthritis–associated interstitial lung disease in European ancestry
Source: Rheumatol Adv Pract. 2024 Jun 4;8(2):rkae059. doi: 10.1093/rap/rkae059 (PMC11157136; doi:10.1093/rap/rkae059)
Supplement: rkae059_Supplementary_Data [file rkae059_supplementary_data.docx]

**SUPPLEMENTARY MATERIALS**

**Supplementary Table S1.** Results of the comparison of the characteristics of the cases and the controls included in the study…………………………………………………………………………………………….. page 2

**Supplementary Table S2.** Results of the genetic case-control association studies restricted to ever smokers……………………………………………………………………………………………………………………………… page 3

**Supplementary Table S3.** Results of the genetic case-control association studies restricted to never smokers……………………………………………………………………………………………………………………………… page 4

**Supplementary Table S1. Results of the comparison of the characteristics of the cases and the controls included in the study**

|  | **Combined** | | | **France** | | | **US** | | | **The Netherlands** | | |
| --- | --- | --- | --- | --- | --- | --- | --- | --- | --- | --- | --- | --- |
|  | **RA-ILD**  **cases** | **RA-noILD controls** | ***P*** | **RA-ILD  cases** | **RA-noILD controls** | ***P*** | **RA-ILD cases** | **RA-noILD controls** | ***P*** | **RA-ILD cases** | **RA-noILD controls** | ***P*** |
|  | n=322 | n=561 |  | n=192 | n=256 |  | n=73 | n=213 |  | n=57 | n=92 |  |
| Male Sex, n (%) | 148 (46.0) | 127 (22.7) | 1.4x10^-12^ | 87 (45.3) | 55 (21.5) | 1.4x10^-7^ | 25 (34.2) | 52 (24.4) | 0.10 | 36 (63.2) | 20 (21.7) | 1.1x10^-6^ |
| Ever smoker, n (%) | 209 (66.3) | 310 (56.2) | 0.003 | 115 (61.5) | 130 (52.2) | 0.053 | 50 (68.5) | 126 (59.4) | 0.17 | 44 (80.0) | 54 (59.3) | 0.01 |
| Mean age at RA onset, year ± sd | 55.0 ± 13.0 | 47.3 ± 14.4 | 4.4x10^-13^ | 55.0 ± 13.7 | 44.0 ± 12.8 | 1.5x10^-13^ | 53.0 ± 12.0 | 50.2 ± 15.8 | 0.17 | 57.3 ± 11.8 | 49.7 ± 13.1 | 9.6x10^-4^ |
| Mean age at index date*, year ± sd | 63.3 ± 10.6 | 61.2 ± 13.1 | 0.02 | 63.6 ± 10.0 | 57.4 ± 12.5 | 5.1x10^-7^ | 61.5 ± 11.7 | 61.8 ± 12.1 | 0.87 | 64.5 ± 10.5 | 70.2 ± 12.3 | 0.006 |
| Mean RA duration at index date*, year ± sd | 8.5 ± 10.5 | 13.9 ± 10.5 | 9.8x10-^12^ | 8.8 ± 10.7 | 13.6 ± 8.3 | 2.6x10^-6^ | 8.5 ± 11.3 | 11.6 ± 12.8 | 0.07 | 7.4 ± 8.8 | 20.6 ± 6.3 | 2.7x10^-11^ |
| UIP, n (%) | 143 (49.3) | - | - | 97 (59.1) | - | - | 18 (26.1) | - | - | 28 (49.1) | - | - |
| *MUC5B* rs35705950, MAF | 0.27 | 0.10 | 1.2x10^-16^ | 0.28 | 0.12 | 3.7x10^-9^ | 0.25 | 0.09 | 4.6x10^-5^ | 0.24 | 0.07 | 1.7x10^-4^ |
| *RPA3-UMAD1* rs12702634, MAF | 0.14 | 0.12 | 0.12 | 0.12 | 0.13 | 0.90 | 0.15 | 0.13 | 0.37 | 0.17 | 0.07 | 0.004 |

*index date is the date of ILD diagnosis for RA-ILD cases, the date of the last normal chest HRCT scan for RA-noILD controls from France and the US, or the date of record review for RA-noILD controls from the Netherlands.

HRCT: high-resolution computed tomography, ILD: interstitial lung disease, MAF: minor allele frequency, RA: rheumatoid arthritis, UIP: usual interstitial pneumonia. *P*-value were obtained by univariate logistic regression.

**Supplementary Table S2. Results of the genetic case-control association studies restricted to ever smokers.**

|  | **France (n=245)** | | **US (n=176)** | | **The Netherland (n=98)** | | **Combined (n=519)** | |
| --- | --- | --- | --- | --- | --- | --- | --- | --- |
|  | **Adjusted** | | **Adjusted** | | **Adjusted** | | **Adjusted** | |
|  | **OR [95 CI]** | ***P*** | **OR [95 CI]** | ***P*** | **OR [95 CI]** | ***P*** | **OR [95 CI]** | ***P*** |
| **RA-ILD vs. RA-noILD** |  |  |  |  |  |  |  |  |
| *MUC5B* rs35705950 | 2.3 [1.3 - 4.1] | 0.005 | 4.5 [2.2 - 9.9] | 9.4x10^-5^ | 4.1 [0.95 - 20.3] | 0.07 | 2.8 [1.9 - 4.2] | 5.2x10^-7^ |
| *RPA3-UMAD1* rs12702634 | 0.8 [0.4 - 1.6] | 0.58 | 1.3 [0.7 - 2.7] | 0.42 | 1.9 [0.4 - 9.6] | 0.41 | 1.2 [0.8 - 1.9] | 0.36 |
| Interaction |  | 0.09 |  | 0.09 |  | 0.99 |  | 0.74 |
| **RA-UIP vs. RA-noILD** |  |  |  |  |  |  |  |  |
| *MUC5B* rs35705950 | 2.6 [1.3 - 5.2] | 0.007 | 12.9 [3.4 - 64.5] | 4.3x10^-4^ | 9.3 [1.6 - 81.1] | 0.02 | 4.2 [2.5 - 7.2] | 8.6x10^-8^ |
| *RPA3-UMAD1* rs12702634 | 0.9 [0.4 - 2.0] | 0.80 | 1.3 [0.3 - 4.2] | 0.69 | 3.5 [0.6 - 23.4] | 0.17 | 1.3 [0.8 - 2.3] | 0.28 |
| Interaction |  | 0.02 |  | 0.99 |  | 0.99 |  | 0.51 |
| **Non-UIP RA-ILD vs. RA-noILD** |  |  |  |  |  |  |  |  |
| *MUC5B* rs35705950 | 1.9 [0.9 - 4.0] | 0.09 | 3.2 [1.4 - 7.7] | 0.008 | 1.9 [0.3 - 13.1] | 0.52 | 2.0 [1.2 - 3.3] | 0.006 |
| *RPA3-UMAD1* rs12702634 | 0.6 [0.2 - 1.7] | 0.40 | 1.05 [0.4 - 2.4] | 0.91 | 1.5 [0.1 - 14.8] | 0.71 | 1.0 [0.6 - 1.8] | 0.94 |
| Interaction |  | 0.31 |  | 0.49 |  | 0.99 |  | 0.81 |
| **RA-UIP vs. non-UIP RA-ILD** |  |  |  |  |  |  |  |  |
| *MUC5B* rs35705950 | 1.5 [0.7 - 3.2] | 0.30 | 4.0 [1.07 - 18.7] | 0.049 | 3.8 [1.1 - 15.8] | 0.04 | 2.0 [1.2 - 3.6] | 0.01 |
| *RPA3-UMAD1* rs12702634 | 1.7 [0.6 - 5.7] | 0.36 | 1.4 [0.3 - 6.3] | 0.64 | 2.0 [0.5 - 9.1] | 0.36 | 1.5 [0.8 - 3.1] | 0.24 |
| Interaction |  | 0.28 |  | 0.71 |  | 0.63 |  | 0.39 |

Multivariable results are adjusted on sex, age at RA onset, age at ILD onset or certified absence of ILD, tobacco smoking status, and country of origin. They are presented with the P-value of the interaction between *MUC5B* rs35705950 and *RPA3-UMAD1* rs12702634.

ILD: interstitial lung disease, OR [95CI]: odd ratio with their 95% confidence interval, RA: rheumatoid arthritis, UIP: usual interstitial pneumonia.

**Supplementary Table S3. Results of the genetic case-control association studies restricted to never smokers**

Multivariable results are adjusted on sex, age at RA onset, age at ILD onset or certified absence of ILD, tobacco smoking status, and country of origin. They are presented with the P-value of the interaction between *MUC5B* rs35705950 and *RPA3-UMAD1* rs12702634.

ILD: interstitial lung disease, OR [95CI]: odd ratio with their 95% confidence interval, RA: rheumatoid arthritis, UIP: usual interstitial pneumonia.

|  | **France (n=191)** | | **US (n=109)** | | **The Netherland (n=48)** | | **Combined (n=348)** | |
| --- | --- | --- | --- | --- | --- | --- | --- | --- |
|  | **Adjusted** | | **Adjusted** | | **Adjusted** | | **Adjusted** | |
|  | **OR [95 CI]** | ***P*** | **OR [95 CI]** | ***P*** | **OR [95 CI]** | ***P*** | **OR [95 CI]** | ***P*** |
| **RA-ILD vs. RA-noILD** |  |  |  |  |  |  |  |  |
| *MUC5B* rs35705950 | 3.0 [1.6 - 5.7] | 4.8x10^-4^ | 2.3 [0.95 - 5.6] | 0.06 | 20.2 [2.0 - 465.2] | 0.02 | 3.1 [1.9 - 5.1] | 3.1x10^-6^ |
| *RPA3-UMAD1* rs12702634 | 1.2 [0.6 - 2.4] | 0.55 | 0.9 [0.3 - 2.4] | 0.89 | 1.9 [0.2 - 16.1] | 0.56 | 1.2 [0.7 - 2.0] | 0.47 |
| Interaction |  | 0.27 |  | 0.48 |  | 0.99 |  | 0.32 |
| **RA-UIP vs. RA-noILD** |  |  |  |  |  |  |  |  |
| *MUC5B* rs35705950 | 6.2 [2.7 - 15.8] | 4.7x10^-5^ | 3.7 [0.9 - 17.0] | 0.07 | 2.7 [0.1 - 126.2] | 0.57 | 5.1 [2.7 - 10.1] | 8.4x10^-7^ |
| *RPA3-UMAD1* rs12702634 | 1.4 [0.6 - 3.3] | 0.46 | 0.9 [0.05 - 5.3] | 0.92 | 0.2 [0.0002 - 12.4] | 0.49 | 1.3 [0.6 - 2.6] | 0.46 |
| Interaction |  | 0.55 |  | 0.99 |  | 0.99 |  | 0.94 |
| **Non-UIP RA-ILD vs. RA-noILD** |  |  |  |  |  |  |  |  |
| *MUC5B* rs35705950 | 1.7 [0.6 - 4.3] | 0.29 | 2.1 [0.7 - 5.8] | 0.17 | 34.2 [2.6 - 1339.6] | 0.02 | 2.2 [1.2 - 4.2] | 0.01 |
| *RPA3-UMAD1* rs12702634 | 0.9 [0.3 - 2.5] | 0.91 | 0.8 [0.2 - 2.3] | 0.69 | 3.2 [0.3 - 32.4] | 0.31 | 0.98 [0.65 - 1.9] | 0.96 |
| Interaction |  | 0.30 |  | 0.99 |  | 0.99 |  | 0.18 |
| **RA-UIP vs. non-UIP RA-ILD** |  |  |  |  |  |  |  |  |
| *MUC5B* rs35705950 | 5.5 [1.6 - 24.6] | 0.01 | 1.2 [0.1 - 10.8] | 0.86 | 0.8 [0.02 - 68.3] | 0.92 | 3.1 [1.3 - 7.9] | 0.01 |
| *RPA3-UMAD1* rs12702634 | 2.0 [0.5 - 9.4] | 0.34 | 0.8 [0.03 - 12.5] | 0.90 | 0.02 [0.0001 - 3.6] | 0.26 | 1.4 [0.5 - 4.0] | 0.49 |
| Interaction |  | 0.89 |  | 0.99 |  | 0.99 |  | 0.39 |
